# Objective Evaluation of Gait Asymmetries in Traditional Racehorses During Pre-Race Inspection: Application of a Markerless AI System in Straight-Line and Lungeing Conditions

**DOI:** 10.3390/ani15121797

**Published:** 2025-06-18

**Authors:** Federica Meistro, Maria Virginia Ralletti, Riccardo Rinnovati, Alessandro Spadari

**Affiliations:** Department of Veterinary Medical Sciences, University of Bologna, Via Tolara di Sopra 50, 40064 Ozzano dell’Emilia, Italy; virginia.ralletti@unibo.it (M.V.R.); riccardo.rinnovati2@unibo.it (R.R.); alessandro.spadari@unibo.it (A.S.)

**Keywords:** pre-race veterinary inspection, objective gait assessment, artificial intelligence, racehorses, traditional racing events

## Abstract

Subtle locomotor asymmetries are common in horses and may go unnoticed during routine pre-race clinical inspections, particularly when based solely on subjective evaluation. This study aimed to describe vertical head and pelvic movement asymmetries in racehorses that passed official pre-race inspections at a traditional racing event. Twenty-four horses were analysed using a markerless AI-based gait analysis system while trotting in-hand and during lungeing in both directions. Asymmetry parameters (HDmin, HDmax, PDmin, and PDmax) were extracted from video recordings, with values ≥0.5 considered clinically relevant. Vertical asymmetries were detected in 71% of horses during straight-line evaluation and in 79% during at least one lungeing direction. Some horses showed relevant asymmetries only under specific movement conditions, underscoring the complementary role of straight-line and lungeing assessments in comprehensive gait evaluation. These results suggest that objective gait analysis could enhance pre-race veterinary assessments, especially in traditional racing, where horses are subjected to significant biomechanical stress, including variable surface properties and repetitive directional loading. In such complex and dynamic environments, relying solely on visual assessment may result in the underdiagnosis of subtle locomotor alterations. The AI-based tools offer potential to improve the detection of subtle irregularities and support evidence-based decisions in performance horse management. Further investigations are warranted to validate the clinical relevance of currently adopted asymmetry thresholds, refine their diagnostic value, and support their integration into standardized pre-race evaluation protocols.

## Simple Summary

Pre-race veterinary inspections aim to ensure that horses are fit to compete, but subtle gait abnormalities may go unnoticed during routine evaluations. This study tested a smartphone-based, artificial intelligence (AI) system to detect movement asymmetries in racehorses at a traditional racing event. Horses were evaluated at the trot, both in straight-line trotting and while lungeing. The system detected vertical asymmetries in many horses, and in some cases, relevant findings were visible only under one specific movement condition. Evaluating horses in pre-race inspection under different movement conditions is crucial, as each may expose unique aspects of gait; findings in one condition may not appear in another, making both straight-line and lungeing assessments complementary rather than interchangeable. The use of AI-based technologies could support early identification of locomotor abnormalities and provide valuable data during pre-race evaluations, aiding veterinarians in protecting horse welfare and performance.

## 1. Introduction

Lameness remains the leading cause of poor performance in horses and presents significant diagnostic challenges, especially when clinical signs are subtle [1,2,3]. Subclinical locomotor asymmetries may precede overt lameness and are often undetected during standard visual evaluations, despite their potential to affect performance or indicate early musculoskeletal dysfunction [3,4].

Subjective lameness evaluations, typically performed by observing the horse trotting in a straight line and on a circle, remain the clinical standard [5]. Straight-line trotting often represents the basis for initial clinical evaluation, offering a consistent setting to assess stride symmetry and regularity [5,6,7]. Lungeing provides complementary information. The biomechanical adaptations required during circular motion, such as increased loading on the outer limb, and axial digital joint rotation, which may influence symmetry patterns and reveal subtle asymmetries not detectable on a straight line [7,8]. Vertical displacement asymmetries are known to increase during lungeing [3,9,10,11]. These variations are influenced by multiple factors, including surface type [8], torso orientation [12], speed, circle radius [13], and rider influence [14]. This complexity supports the inclusion of both straight and lungeing evaluation into comprehensive gait assessment protocols, particularly in performance horses.

Despite being the standard practice [9,10,15], subjective evaluations suffer from considerable inter- and intra-observer variability, especially in mild or hindlimb lameness [16,17]. To overcome these limitations, objective tools such as inertial sensors and optical motion capture have been developed [18,19,20], but their use in the field is limited by setup complexity and time constraints [20,21].

Recently, a novel objective markerless artificial intelligence (AI)-based gait analysis system for movement analysis has been introduced. This system uses computer vision technology, a subset of AI, which is specifically trained to identify and track the pelvis, head, and hooves in the video stream of trotting horse without requiring attached sensors or reflective markers [20,21,22,23]. A mean difference of 2.2 mm in head and pelvic movement was reported when comparing this AI-based system to a multi-camera optical motion capture system [23].

Several studies using objective motion analysis tools have reported a high prevalence of vertical asymmetries exceeding clinical thresholds, up to 90%, even in horses deemed sound, raising questions about whether these asymmetries reflect subclinical pathology or individual biomechanical variation [3,7,10,13,24,25]. In this context, assessing horses considered “fit to race” may help clarify the functional relevance of mild asymmetries in high-performing equine athletes.

Racehorses competing in traditional racing events, such as the Palio, are exposed to intense biomechanical stress [26]. This population offers a valuable model for evaluating movement asymmetries in real-world conditions. Pre-race evaluations typically aim to identify overt lameness [25,27], but more subtle locomotor abnormalities often remain undetected [26]. Investigating the prevalence and nature of motion asymmetries in horses considered “fit to race” may help clarify the functional significance of mild asymmetries and support the implementation of preventive strategies. This study aimed to investigate the presence and distribution of vertical movement asymmetries in racehorses considered clinically sound during routine pre-race veterinary examinations. By describing the magnitude and frequency of head and pelvic asymmetries detected using a markerless AI-based gait analysis system, we sought to characterize the asymmetry profiles observed in this specific population under straight-line trot and lungeing conditions. Particular attention was given to evaluating how different movement tasks contributed to the detection of clinically relevant asymmetries, and whether certain conditions revealed findings that were not apparent in others. Ultimately, this study aimed to explore the feasibility and potential added value of using objective gait analysis in the context of field-based veterinary assessments.

## 2. Materials and Methods

### 2.1. Study Design and Setting

This was a prospective observational study conducted during pre-race veterinary evaluations at a traditional racing event (Palio of Faenza, Faenza, (RA), Italy). The aim was to assess gait asymmetries in racehorses under routine field conditions using an objective markerless AI-based gait analysis system. All data were collected without modifying the standard clinical procedures required for pre-race fitness-to-compete examination. Horses were evaluated in both straight-line and lungeing conditions, allowing within-subject comparisons of movement asymmetry.

### 2.2. Study Population

The study population consisted of 24 racehorses actively competing in the Palio of Faenza racing event. All horses were stabled in a single training facility and followed similar training regimens. The group included 20 Thoroughbreds and 4 Anglo-Arabians, with ages ranging from 4 to 13 years (mean: 7 years). Sex distribution was as follows: 13 geldings and 11 females. Horses were recruited at the time of the mandatory pre-race veterinary inspection.

Prior to inclusion in the study, all horses underwent through a general physical examination performed by equine clinicians at the Equine Surgery Department, Faculty of Veterinary Medicine, University of Bologna.

Inclusion in the study required that horses successfully pass the general clinical examination performed as part of the official fitness-to-compete assessment. Horses presenting with relevant clinical abnormalities during the general evaluation were not included in the study.

Informed consent was obtained from all owners prior to participation. 

Ethical approval was not required, as the study was non-invasive and conducted entirely within the framework of standard clinical practice, in accordance with the University of Bologna’s regulatory guidelines.

### 2.3. Clinical Evaluation of Lameness and Trotting Conditions

Each horse underwent a standard pre-race orthopaedic evaluation performed by two experienced equine veterinarians (each with more than 10 years of experience in equine orthopaedics). The examination protocol included palpation of the distal limbs, axial skeleton, and back, followed by flexion tests and dynamic in-hand evaluation at the trot.

During the dynamic evaluation, horses were trotted in-hand over a flat, soft surface approximately 30 m long, typical of the trot-up area used for pre-race assessments at the training facility. This allowed the collection of approximately 25 contiguous strides per pass. Lungeing was subsequently performed in both directions (left and right reins) on a soft footing surface, using a circle with a diameter of 12 to 15 m. Horses were lunged for a minimum of 45 s per direction to ensure sufficient stride collection for analysis [22].

The subjective evaluation of gait was based on the American Association of Equine Practitioners (AAEP) lameness grading scale (0–5) [28], and scores were recorded during the clinical examination. Simultaneously, a third veterinarian recorded video footage of the horse’s movement using a smartphone mounted on a handheld stabilizer for subsequent objective gait analysis. The video recordings were obtained during the in-hand trot and both lungeing directions, without interfering with the standard clinical workflow.

Although the two surfaces (straight-line and lungeing) were not identical, both were categorized as “soft,” providing consistent support for gait assessment. The same setup and protocol were followed for all horses to ensure repeatability and minimize variability due to environmental conditions.

### 2.4. Instrumentation

A markerless AI-based gait analysis system (Sleip AI^®^, Uppsala, Sweden, version 1.0.10) was used for data collection. Video recordings were acquired using an iPhone^®^ 14 Pro mounted on a handheld stabilizer to ensure image stability and maintain the horse within the camera frame throughout the evaluation. During the straight-line trotting, the operator stood approximately 3 m behind the starting point of the 30-m track and recorded the horse straight from behind, with the horse trotting away from the camera, and then toward [22]. For the lungeing evaluations, the operator positioned themselves approximately 10 m outside the 12–15 m lungeing circle, as recommended by the software provider. Care was taken to keep the entire horse within the field of view during all phases of the evaluation [22]. No physical markers or wearable sensors were applied to the horses. The software identifies anatomical landmarks based on deep learning algorithms trained on large datasets of equine movement patterns [23]. All videos were recorded offline during the evaluation sessions and subsequently uploaded to the markerless AI-based gait analysis system platform for processing. No real-time analysis was performed in the field.

### 2.5. Data Processing

All video recordings were uploaded to the markerless AI-based gait analysis system cloud-based platform for processing. The software uses neural network-based motion tracking to identify and follow anatomical landmarks without the need for physical markers. Each recording was automatically analysed to extract key asymmetry parameters associated with vertical head and pelvic displacement, including HDmin, HDmax, PDmin, and PDmax. These correspond to the differences between the two minima and maxima in vertical displacement during each stride cycle and are used to quantify asymmetries related to impact (minima) and push-off (maxima).

Only recordings meeting predefined technical standards were included in the analysis to ensure reliability. Specifically, each session had to include a minimum of 10 analysable strides for the forelimbs and 10 for the hindlimbs, as recommended by the system to produce validated results. Recordings were reviewed for quality, and those in which the horse did not remain fully visible throughout the movement, or where motion artifacts interfered with limb detection, were excluded. All 24 horses included in the study yielded at least one complete and analysable video for each condition (straight-line, left circle, and right circle).

Asymmetry magnitude is initially computed as a percentage of the vertical range of motion (RoM) for each stride and is then converted by the software into a normalized score on a scale from 0 to 2, allowing for standardized comparisons across recordings. Thresholds for asymmetry severity were as follows: very mild (0.2–0.49), mild (0.5–0.9), moderate (1.0–1.49), and severe 1.5–2.0). In this study, only horses with at least mild asymmetry (≥0.5) were considered to have clinically relevant motion asymmetries. This cut-off was based on thresholds reported in previous studies using this gait analysis system for gait monitoring and lameness detection [25] and reflects the level at which asymmetry becomes visually evident to the trained human eye. It does not represent a diagnostic criterion, nor a definition of normality. Importantly, the term “asymmetry” here refers exclusively to objective deviations in vertical motion and does not imply clinical lameness. In the presence of bilateral asymmetries (e.g., impact asymmetry on one limb and push-off asymmetry on the contralateral limb), the limb with the highest asymmetry score was identified as the primary asymmetric limb.

Asymmetry values were exported as absolute (unsigned) numerical values for each limb (LF, RF, LH, and RH) and did not indicate side preference unless specifically analysed. No recordings were discarded due to insufficient stride count or poor video quality among the selected datasets.

### 2.6. Statistical Analysis

All data were compiled in Microsoft Excel and analysed using R software (version 4.3.2; R Foundation for Statistical Computing, Vienna, Austria). For each horse and for each movement condition (straight-line trot, left circle, and right circle), the following parameters were extracted from the gait analysis system: number of analysed strides, HDmin, HDmax, PDmin, and PDmax, and the respective standard deviations (SD) for each limb (left fore, right fore, left hind, and right hind).

Asymmetry values were expressed as absolute (positive) values for statistical calculations, while preserving the original limb assignment to allow identification of the most asymmetric limb per condition. A total asymmetry score was calculated for each horse under each condition as the sum of the asymmetry scores across all four limbs.

Descriptive statistics (mean, standard deviation, median, minimum, maximum, and interquartile range [Q1–Q3]) were calculated for each asymmetry parameter and condition.

The prevalence of clinically relevant asymmetry (defined as ≥0.5) was calculated for each condition. Chi-square (χ^2^) tests were used to compare prevalence rates among conditions. In addition, the limb with the highest asymmetry was identified for each horse and each condition, and the frequency of left-sided versus right-sided asymmetry was analysed using an exact binomial test to evaluate potential lateralization patterns.

Exploratory analyses were also conducted to assess potential associations between signalment and asymmetry. The total asymmetry score was considered under each movement condition. Normality testing indicated that age was not normally distributed under any condition (Shapiro–Wilk test, *p* = 0.050). Total asymmetry scores were normally distributed under straight-line (*p* = 0.767) and right-circle (*p* = 0.212) conditions but not under the left-circle condition (*p* = 0.004). Given the borderline or non-normal distribution of age, Spearman’s rank correlation coefficient was used for all conditions. For group comparisons, horses were categorized by sex (male vs. female) and breed (Thoroughbred vs. Anglo-Arabian). Data normality was tested using the Shapiro–Wilk test, and comparisons were performed using the Mann–Whitney U test due to non-normal distribution in at least one group.

Statistical significance was set at *p* < 0.05 for all analyses.

No formal sample size calculation was performed, as this was an exploratory field study including all eligible horses available during the data collection period.

## 3. Results

All 24 horses included in the study were declared clinically fit to race following the routine pre-race veterinary examinations. Objective gait data were successfully recorded for all horses under each movement condition, and no video recordings were excluded due to technical issues or stride count insufficiency.

The number of analysed strides per condition is summarized in Table 1. Horses trotted in a straight line generally produced fewer forelimb and hindlimb strides compared to circular assessments.

Individual asymmetry values per horse and condition are illustrated in Supplementary Figures S1–S3 and Tables S1, S3, and S5, providing a detailed distribution of vertical head and pelvic asymmetries.

Descriptive statistics were calculated for each asymmetry parameter (HDmin, HDmax, PDmin, and PDmax) and for each limb (LF, RF, LH, and RH) under all three movement conditions. These data are reported in Supplementary Tables S2, S4, S6, and S7.

Asymmetries of clinically relevant magnitude (i.e., values ≥ 0.5 in at least one of the four vertical displacement parameters: HDmin, HDmax, PDmin, or PDmax) were observed in the majority of horses. A horse was considered to show clinically relevant asymmetry in a given condition (straight line, right circle, or left circle) if at least one parameter in any limb reached or exceeded the 0.5 threshold. Specifically, 17/24 horses (71%) exceeded the threshold on the straight line, 15/24 (63%) on the right circle, and 19/24 (79%) on the left circle.

**Figure 1:**
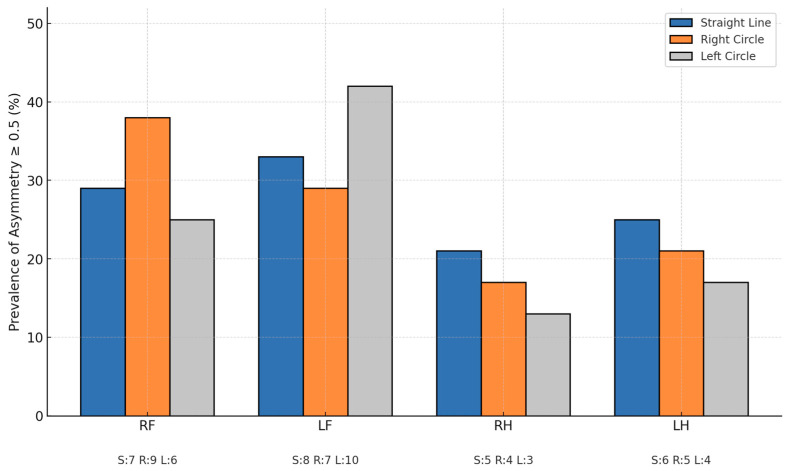
Prevalence (%) of clinically relevant asymmetries (≥0.5) per limb across different movement conditions (n = 24). The graph illustrates the percentage of horses exceeding the asymmetry threshold in the right forelimb (RF), left forelimb (LF), right hindlimb (RH), and left hindlimb (LH), when trotting on a straight line (S), on the right circle (R), and on the left circle (L). Each bar corresponds to a specific movement condition, with colours indicating straight-line (blue), right circle (orange), and left circle (green). Absolute numbers of affected horses are indicated below each column label for each condition.

To summarize asymmetry severity, we classified each horse in each condition based on the highest single asymmetry value observed among all limbs and parameters. Severity was categorized as follows: very mild (0.2–0.49), mild (0.5–0.99), moderate (1.0–1.49), and severe (1.5–2.0). On the straight line, 12/24 horses were classified as very mild, 5/24 as mild, 5/24 as moderate, and 2/24 as severe. On the right circle, 11/24 were very mild, 10/24 mild, 2/24 moderate, and 1/24 severe. On the left circle, 14/24 were very mild and 10/24 mild, with no horses classified as moderate or severe (Figure 1 and Table 2)

There was no statistically significant difference in the prevalence of clinically relevant asymmetries (≥0.5) between movement conditions (χ^2^ = 1.61, df = 2, *p* = 0.446). No significant lateralization pattern was observed in any condition. On the straight line, 12 horses had greater asymmetry on the left and 10 on the right (binomial test, *p* = 0.832); on the right circle, 11 were left-sided and 9 right-sided (*p* = 0.824); and on the left circle, 14 were left-sided and 6 right-sided (*p* = 0.115).

Clinically relevant asymmetries (≥0.5) were observed exclusively in one movement condition in several horses: 4/24 on the straight line, 3/24 on the right circle, and 5/24 on the left circle (Table 3).

**Figure 2:**
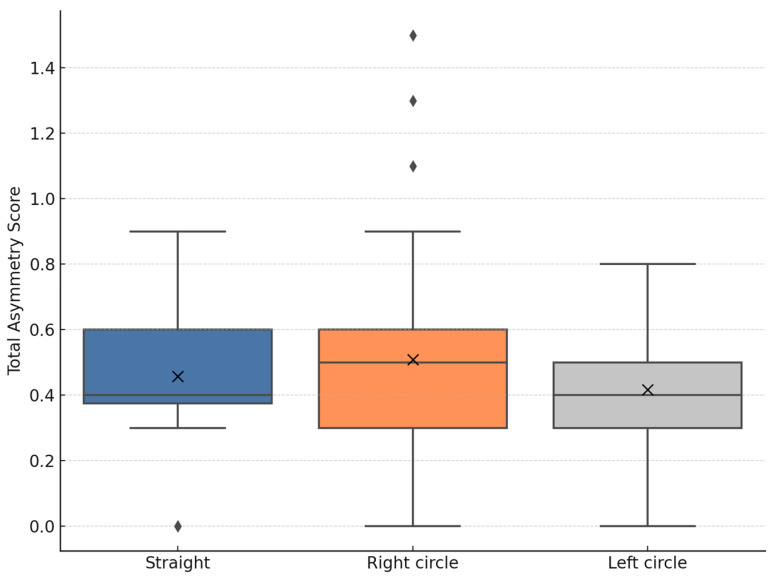
Total asymmetry scores across the three evaluated movement conditions. Boxplots represent the distribution of asymmetry values among 24 horses. The box represents the interquartile range (IQR), the horizontal line indicates the median, whiskers extend to 1.5× IQR, and black dots indicate outliers. ‘X’ symbols represent the mean.

Median total asymmetry was 0.40 on the straight line, 0.50 on the right circle, and 0.40 on the left circle. The highest variability in scores was observed under right-circle conditions (mean ± SD: 0.51 ± 0.39), followed by straight line (0.46 ± 0.21) and left circle (0.42 ± 0.17) (Figure 2).

No significant correlation was found between age and total asymmetry score in any of the movement conditions: straight line (ρ = –0.114, *p* = 0.596); right circle (ρ = –0.184, *p* = 0.390), and left circle (ρ = 0.042, *p* = 0.845). Data are provided in Supplementary Table S8.

Mann–Whitney U tests revealed no statistically significant differences in total asymmetry scores between breeds or sexes across all conditions: Breed: *p* = 0.613 (straight-line), 0.938 (right circle), 0.751 (left-circle); and Sex: *p* = 0.954 (straight line), 0.123 (right circle), 0.594 (left circle). Data are provided in Supplementary Tables S9 and S10, and in Figure S4.

## 4. Discussion

This study described vertical motion asymmetries in a population of racehorses evaluated during routine pre-race veterinary inspections at a traditional racing event, using a markerless AI-based gait analysis system. All horses were deemed “fit to race” through standard clinical evaluation, offering a valuable opportunity to explore the occurrence and distribution of asymmetries in presumed sound performance horses under field conditions. To our knowledge, this is the first study applying such technology to horses competing in traditional racing events like the Palio, where asymmetrical locomotor environments, sharp directional changes, and irregular ground conditions can significantly affect locomotor patterns [26,27,29].

The findings showed a high prevalence of vertical asymmetries across all tested conditions, with 79% of horses exceeding the ≥0.5 threshold during lungeing and 71% during straight-line evaluation. These results support previous studies reporting frequent subclinical asymmetries in horses considered sound by their owners or veterinarians [9,11,24,30]. Descriptive statistics of HDmin, HDmax, PDmin, and PDmax across all limbs and conditions showed that the highest asymmetry value varied between horses and movement tasks. This highlights the heterogeneous expression of movement asymmetries, both in terms of affected limb and gait phase (impact vs. push-off). Additionally, in our population, 20.8% of horses showed relevant asymmetry exclusively during left-circle lungeing and 16.7% exclusively on the straight line. The detection of condition-specific asymmetries suggests that relying on assessment on a single movement condition may underestimate clinically relevant asymmetries and that locomotor expression is influenced by the specific demands of each movement task [5,31]. These discrepancies likely stem from distinct mechanical demands and proprioceptive input during each gait context. Horses may adopt different movement strategies under straight or circular loading, particularly on variable surfaces [7,8,13,32].

Our data further revealed that asymmetries during lungeing often involved the inside forelimb, in line with previous biomechanical studies suggesting a possible reflection of circle-induced axial loading and compensatory adaptations [7,8]. Notably, right-forelimb asymmetries during right-circle trotting occurred significantly more often than during straight-line assessment. However, it is important to recognize, as highlighted by prior authors [3,8,10], that vertical asymmetry does not necessarily equate to clinical lameness and should be interpreted with caution.

Although age, sex, and breed are commonly considered potential contributors to locomotor asymmetries, our analysis did not reveal any statistically significant associations [9]. The absence of correlation between signalment and total asymmetry score suggests that the subtle vertical motion differences observed in this group may be independent of these demographic variables. However, it is important to acknowledge the limited number of Anglo-Arabians and the overall small sample size, which may have reduced the statistical power to detect meaningful differences.

From a technical standpoint, the markerless AI-based gait analysis system showed improved data acquisition during lungeing, as it allowed continuous lateral tracking of both head and pelvic motion [20,22]. This contrasts with straight-line recordings, where hindlimb analysis depends on tracking the horse trotting away from the camera, requiring multiple passes to obtain reliable data.

Events such as the Palio of Faenza add originality and practical significance. Horses competing in traditional events face distinct locomotor challenges, including uneven grass tracks, abrupt directional shifts, and irregular footing, that may exacerbate subtle asymmetries [26,33,34]. While asymmetries in such settings may reflect adaptive rather than pathological responses, they still warrant attention due to their potential role in injury risk over time. Surface characteristics are a known modifier of locomotor mechanics, and the clinical interpretation of motion data must account for footing, direction, and horse-specific biomechanics [3,8,29]. The Palio of Faenza is held on a 154-m horseshoe-shaped track specifically designed for the event. Its semi-circular layout and single-lane configuration impose curved, asymmetric trajectories that increase mechanical stress on the limbs, especially during rapid acceleration and directional transitions. The track surface consists of natural grass and is classified as soft ground, which may further modulate locomotor dynamics during high-speed motion. This context reinforces the clinical value of including circular assessments in gait evaluation protocols, particularly in traditional racing environments [33,34].

Our findings support the growing role of objective motion analysis as a preventive tool for performance management in horses subjected to non-standard training or racing environments, such as traditional racing and classical flat-racing Similar to what is being tested by the British Horseracing Authority [35], integrating AI-based tools into pre-race evaluations could support clinicians in identifying horses with subclinical asymmetries that would benefit from closer monitoring. By providing objective, repeatable, and field-compatible assessments, such tools may support earlier identification of horses at risk, contribute to performance preservation, and help modernize veterinary decision-making under evidence-based standards. The present study aligns with the goals of the updated 2025 Italian Ministry of Health ordinance regulating Pali and Giostre, which emphasizes animal welfare, standardization of pre-race veterinary evaluations, and the potential use of objective technologies to support clinical decision-making [36]. By applying a non-invasive, markerless gait analysis system during the official pre-race inspection, this work demonstrates a practical approach that may enhance transparency, repeatability, and early detection of locomotor irregularities, in line with regulatory recommendations.

Despite the strengths of this research, certain limitations should be acknowledged. The relatively small sample size of 24 horses, although providing valuable insights, may limit the generalizability of the findings. Additionally, including horses with confirmed lameness in future studies could enhance our understanding of how assessments in circles differentiate between compensatory movement patterns and true pathological asymmetries. Finally, although this study standardized surface conditions and circle size, other factors, such as trotting speed, surface type, and handler influence, were not extensively explored and warrant further investigation. A further development of this work could include assessing observer agreement with the markerless AI-based gait analysis system during lungeing, possibly using the same dataset. Future research should address these elements to enhance the interpretability and clinical value of motion analysis in pre-competition evaluations.

## 5. Conclusions

This study documented vertical movement asymmetries in racehorses that had passed the official pre-race veterinary inspection at a traditional racing event. Using a markerless AI-based gait analysis system, we observed a high prevalence of asymmetries, with some horses showing relevant findings only under one movement condition. This suggests that standard trot-up evaluations may miss subtle gait irregularities unless both straight-line and lungeing assessments are performed. Lungeing appeared particularly effective for detecting asymmetries, likely due to the biomechanical demands of circular motion and the improved continuity of data capture. Additionally, our findings highlight the value of objective motion analysis as a complementary tool in field-based pre-race evaluations. The use of markerless AI systems in pre-competition settings, especially those involving irregular surfaces or high mechanical stress, as in traditional races, may support early detection of locomotor asymmetries and inform preventive management strategies. Further studies on larger populations, including horses with known musculoskeletal conditions, are needed to refine asymmetry thresholds and assess the influence of environmental factors on gait evaluation.

## Figures and Tables

**Table 1:** Number of analysed strides per horse during straight-line and circular movement evaluations.

Condition	Forelimb Strides (Mean [Range])	Hindlimb Strides (Mean [Range])
Straight line	30 (15–45)	24 (12–42)
Right circle	42 (21–60)	34 (12–51)
Left circle	37 (18–52)	32 (14–48)

**Table 2:** Distribution of horses according to the severity of vertical asymmetries under each movement condition. Severity was classified by identifying the highest single asymmetry value (among HDmin, HDmax, PDmin, and PDmax) observed across all limbs (LF, RF, LH, and RH) for each horse in each condition: no asymmetry (0–0.2), very mild (0.2–0.49), mild (0.5–0.99), moderate (1.0–1.49), and severe (1.5–2.0).

Condition	No Asymmetry (0–0.2)	Very Mild (0.2–0.49)	Mild (0.5–0.99)	Moderate (1.0–1.49)	Severe (1.5–2.0)
Straight Line	0	12	5	5	2
Right Circle	0	11	10	2	1
Left Circle	0	14	10	0	0

**Table 3:** Distribution of horses with clinically relevant asymmetry (≥0.5) identified in a single movement condition. Horses included in this table exceeded the asymmetry threshold in one movement condition only, with values remaining below the threshold in the other two.

Condition	Horses with Exclusive Relevant Asymmetry	%
Straight Line only	4	16.7
Right Circle only	3	12.5
Left Circle only	5	20.8

## Data Availability

The data supporting the findings of this study are not publicly available due to privacy and institutional restrictions but may be provided by the corresponding author upon reasonable request.

## Supplementary Materials

The following supporting information can be downloaded at: https://www.mdpi.com/article/10.3390/ani15121797/s1, Detailed Bland-Altman and Lin’s Concordance Correlation plots. Table S1: Raw data for vertical displacement asymmetries in straight-line trotting; Table S2: Descriptive statistics for vertical displacement asymmetries (absolute values) in straight line trotting; Table S3: Raw data for vertical displacement asymmetries in right-circle trotting; Table S4: Descriptive statistics for vertical displacement asymmetries (absolute values) in left circle lungeing; Table S5: Raw data for vertical displacement asymmetries in straight-line trotting; Table S6: Descriptive statistics for vertical displacement asymmetries (absolute values) in left circle lungeing; Table S7: Descriptive Statistics of Limb Asymmetries; Table S8: Descriptive statistics of total asymmetry scores stratified by breed (Thoroughbred vs Anglo-Arabian) for each movement condition; Table S9: Descriptive statistics of total asymmetry scores stratified by sex (male vs female) for each movement condition; Table S10: Spearman’s rank correlation coefficient for age correlation; Figure S1. Vertical displacement asymmetry parameters during straight-line trot for each horse (n = 24); Figure S2. Vertical displacement asymmetry parameters during right-circle lungeing for each horse (n = 24); Figure S3. Vertical displacement asymmetry parameters during left-circle lungeing for each horse (n = 24); Figure S4: Scatterplot of horse age versus total asymmetry score across movement conditions (n = 24).
