# Supplementary material for: Objective Evaluation of Gait Asymmetries in Traditional Racehorses During Pre-Race Inspection: Application of a Markerless AI System in Straight-Line and Lungeing Conditions
Source: Animals (Basel). 2025 Jun 18;15(12):1797. doi: 10.3390/ani15121797 (PMC12189307; doi:10.3390/ani15121797)
Supplement: Supplementary file 1 [file animals-15-01797-s001.zip › animals-3694144-supplementary.pdf]

Supplementary Matherials

HdMax	HdMin	PdMax	PdMin
0	-0,6	0,3	0
0,6	0	0	0,2
0,3	0	0,3	-0,4
0	0,6	0	0
0	0,4	0,6	0
-0,4	0	0	0
0	0,9	0	-0,3
0,3	0	-0,2	0
0	-0,5	0	-0,4
0,4	-0,4	0	-0,7
0	-0,6	0	-0,6
0	-0,4	0	0
0	0,4	0	-0,3
0	0,4	0	0,4
0	0	0	-0,4
0	-0,3	0	0
0	0	-0,4	0,2
0	-0,3	0	-0,2
0	0	0	0
0	0	-0,5	0
0	0,3	0,6	0
0,8	0	0	0,3
0	0	0	0
0	0	0	0

Table S1: **Raw data for vertical displacement asymmetries in straight-line trotting**. The table reports the original values of peak differences in vertical displacement for each limb segment during lungeing evaluation: HDmax, HDmin, PDmax, and PDmin. Negative values indicate lower displacement in the respective limb compared to the contralateral, as automatically provided by the AI-based motion analysis system.

	HdMax	HdMin	Pdmax	Pdmin
**count**	24.0	24.0	24.0	24.0
**mean**	0.116	0.254	0.120	0.183
**std**	0.225	0.268	0.208	0.2160
**min**	0.0	0.0	0.0	0.0
**25%**	0.0	0.0	0.0	0.0
**50%**	0.0	0.3	0.0	0.1
**75%**	75	0.4	225	325
**max**	0.8	0.9	0.6	0.7

Table S2: Descriptive statistics for vertical displacement asymmetries (absolute values) in straight line trotting. This table presents the descriptive statistics (count, mean, standard deviation, minimum, and quartiles) for the absolute values of HDmax, HDmin, PDmax, and PDmin. Converting all negative values to positive allowed for summarizing the overall magnitude of asymmetry regardless of side, to better reflect the clinical relevance of asymmetry severity rather than direction.

HdMax	HdMin	PdMax	PdMin
0	-0,2	0,1	0
0,8	0	0	0,3
-0,1	0,1	0	0
0,7	0,8	-0,1	-0,1
0.7	0	0,4	-0,4
0	0,2	-0,3	0,1
0,3	1,1	-0,3	0
0,4	0	-0,9	-0,3
0,4	-0,5	0,2	0,1
0,2	-0,4	-0,5	-0,3
-0,2	0	-0,3	-0,3
0,1	0,2	0,2	0
0,5	0,2	0	-0,2
-0,3	-0,1	0	-0,1

0,3	-0,2	-0,5	0,1
0,2	0,5	0,1	-0,3
-0,1	-1,5	0	0,2
-0,5	-0,5	0,4	0
0,4	-0,3	-0,3	-0,2
-0,4	0,2	-0,2	0
0,2	-0,2	0,6	0
1,3	-0,3	0,1	0
0,1	0,2	-0,1	0,1
-0,3	0,1	-0,3	-0,1

Table S3: **Raw data for vertical displacement asymmetries in right-circle trotting**. The table reports the original values of peak differences in vertical displacement for each limb segment during lungeing evaluation: HDmax, HDmin, PDmax, and PDmin. Negative values indicate lower displacement in the respective limb compared to the contralateral, as automatically provided by the AI-based motion analysis system

	HdMax	HdMin	Pdmax	Pdmin
**count**	24.0	24.0	24.0	24.0
**mean**	0.354	325	0.245	0.133
**std**	0.294	0.361	0.224	0.127
**min**	0.0	0.0	0.0	0.0
**25%**	0.175	0.1	0.1	0.0
**50%**	0.3	0.2	0.2	0.1
**75%**	0.425	0.425	0.325	0.225
**max**	1.3	1.5	0.9	0.4

Table S4: Descriptive statistics for vertical displacement asymmetries (absolute values) in left circle lungeing. This table presents the descriptive statistics (count, mean, standard deviation, minimum, and quartiles) for the absolute values of HDmax, HDmin, PDmax, and PDmin. Converting all negative values to positive allowed for

summarizing the overall magnitude of asymmetry regardless of side, to better reflect the clinical relevance of asymmetry severity rather than direction.

HDmax	HDmin	PdMax	PdMin
0	-0,3	0,3	0
0	-0,3	0	0
-0,3	0,3	0	0
0,4	0	0,2	0
0	-0,6	0,4	-0,4
0	-0,4	0	0,2
0	0,7	0	-0,4
0,5	0	0	0
0	0	0,3	-0,3
0	0	-0,2	0
-0,8	0	0	0
0	0	0	0
-0,3	0	0	-0,2
-0,5	0	0,2	-0,2
-0,4	0	0	0
0	0	0	-0,6
0	0,3	-0,3	0,3
0,4	0	0	0
0	-0,3	0	-0,2
0,5	0	0	0
-0,5	0	0	0
-0,5	0	0	0
0	0,3	-0,2	0
-0,5	0	0	0

Table S5: **Raw data for vertical displacement asymmetries in straight-line trotting**. The table reports the original values of peak differences in vertical displacement for each limb segment during lungeing evaluation: HDmax, HDmin, PDmax, and PDmin. Negative values indicate lower displacement in the respective limb compared to the contralateral, as automatically provided by the AI-based motion analysis system.

	HdMax	HdMin	PdMax	PdMin
**count**	24.0	24.0	24.0	24.0
**mean**	0.233	0.145	0.085	0.116
**std**	0.2548	0.212	0.132	0.173
**min**	0.0	0.0	0.0	0.0
**25%**	0.0	0.0	0.0	0.0
**50%**	0.15	0.0	0.0	0.0
**75%**	0.5	0.3	0.2	0.2
**max**	0.8	0.7	0.4	0.6

Table S6: Descriptive statistics for vertical displacement asymmetries (absolute values) in left circle lungeing. This table presents the descriptive statistics (count, mean, standard deviation, minimum, and quartiles) for the absolute values of HDmax, HDmin, PDmax, and PDmin. Converting all negative values to positive allowed for summarizing the overall magnitude of asymmetry regardless of side, to better reflect the clinical relevance of asymmetry severity rather than direction.

Condition	Limb	Mean	Std	Min	Q1	Median	Q3	Max
Straight Line	RF	0,208	0,286	0	0	0	0,4	0,9
Straight Line	LF	0,162	0,224	0	0	0	0,4	0,6
Straight Line	RH	0,121	0,193	0	0	0	0,3	0,6
Straight Line	LH	0,2	0,228	0	0	0,1	0,4	0,7
Right Circle	RF	0,254	0,387	0	0	0	0,425	1,3
Right Circle	LF	0,196	0,341	0	0	0	0,325	1,5
Right Circle	RH	0,104	0,178	0	0	0	0,225	0,6

Right Circle	LH	0,154	0,228	0	0	0	0,3	0,9
Left Circle	RF	0,108	0,204	0	0	0	0,075	0,7
Left Circle	LF	0,258	0,248	0	0	0,3	0,5	0,8
Left Circle	RH	0,108	0,164	0	0	0	0,2	0,6
Left Circle	LH	0,096	0,149	0	0	0	0,2	0,4

Table S7: Descriptive Statistics for Limb Asymmetries
Descriptive statistics of asymmetry scores for each limb (right forelimb [RF], left forelimb [LF], right hindlimb [RH], and left hindlimb [LH]) across all movement conditions (Straight Line, Right Circle, and Left Circle).
For each limb-condition combination, the table reports the mean, standard deviation (SD), minimum, first quartile (Q1), median, third quartile (Q3), and maximum value.
Data were derived from objective gait analysis performed using a markerless AI-based system. All values represent normalized, unitless asymmetry scores.

Condition	Group	Category	n	mean	SD	median	Q1	Q3	Mann-Whitney p
**Straight Line**	Breed	Anglo-Arabian	4	585	0.235	0.645	0.472	0.757	0.3323955309279668
**Straight Line**	Breed	Thoroughbred	20	735	0.131	0.71	0.66	0.8175	0.3323955309279668
**Right Circle**	Breed	Anglo-Arabian	4	0.54	0.140	0.52	0.48	0.580	0.48552728648567456
**Right Circle**	Breed	Thoroughbred	20	0.605	0.213	0.59	0.4425	0.802	0.485
**Left Circle**	Breed	Anglo-Arabian	4	0.63	0.087	0.645	0.6075	0.667	0.277
**Left Circle**	Breed	Thoroughbred	20	0.570	0.089	0.58	0.517	0.642	0.277

Table S8: Descriptive statistics of total asymmetry scores stratified by breed (Thoroughbred vs Anglo-Arabian) for each movement condition.
The table reports the number of horses (n), mean, standard deviation (SD), median, first quartile (Q1), and third quartile (Q3) of total asymmetry scores for each condition (straight line, right circle, left circle).
As data were not normally distributed, the Mann–Whitney U test was used to compare total asymmetry scores between breeds. Corresponding p-values are reported in the last column.

Condition	Group	Category	n	mean	SD	median	Q1	Q3	Mann-Whitney p
**Straight Line**	Sex	Female	11	0.649	0.161	0.7	0.580	735	0.104
**Straight Line**	Sex	Male	13	0.761	0.139	0.76	0.67	0.87	0.104
**Right Circle**	Sex	Female	11	0.550	0.194	0.53	405	0.64	0.223
**Right Circle**	Sex	Male	13	0.631	0.208	0.63	0.54	0.81	0.223
**Left Circle**	Sex	Female	11	0.579	0.103	0.58	515	0.65	0.907
**Left Circle**	Sex	Male	13	0.580	0.081	0.58	0.54	0.64	0.907

Table S9: Descriptive statistics of total asymmetry scores stratified by sex (male vs female) for each movement condition. The number of horses (n), mean, SD, median, Q1, and Q3 are provided for each group.
Mann–Whitney U test results are included to evaluate potential differences in asymmetry scores between sexes across movement conditions.

Condition	Spearman's ρ	p-value
Straight Line	-0,114	0,596
Right Circle	-0,184	0,39
Left Circle	0,042	0,845

Table S10: Spearman's rank correlation coefficient for age correlation: Summary of statistical analyses performed for each movement condition (straight line, right circle, left circle). For each condition, the table reports the results of: **Normality testing** of the age variable (Shapiro–Wilk test); **Spearman's rank correlation coefficient** between age and total asymmetry score. None of the comparisons reached statistical significance.

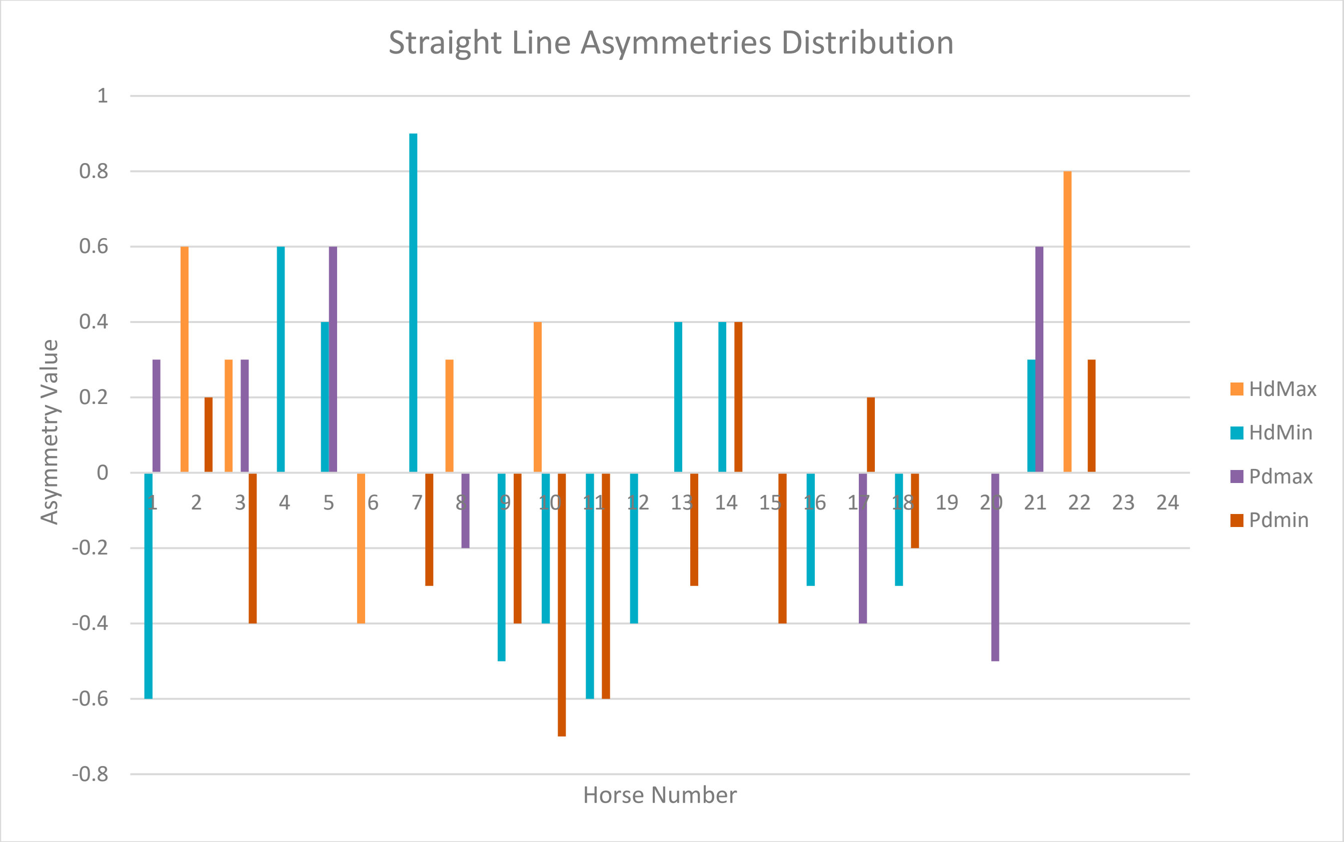

Figure S1. Vertical displacement asymmetry parameters during straight-line trot for each horse (n = 24).

Bar plots show the values of HDmin, HDmax, PDmin, and PDmax for each individual horse, as measured during in-hand trot on a straight line. All horses included in the study are shown, and all asymmetry values (including those below the clinical threshold) are presented. No data are missing; values close to zero may appear small or indistinct on the plot.

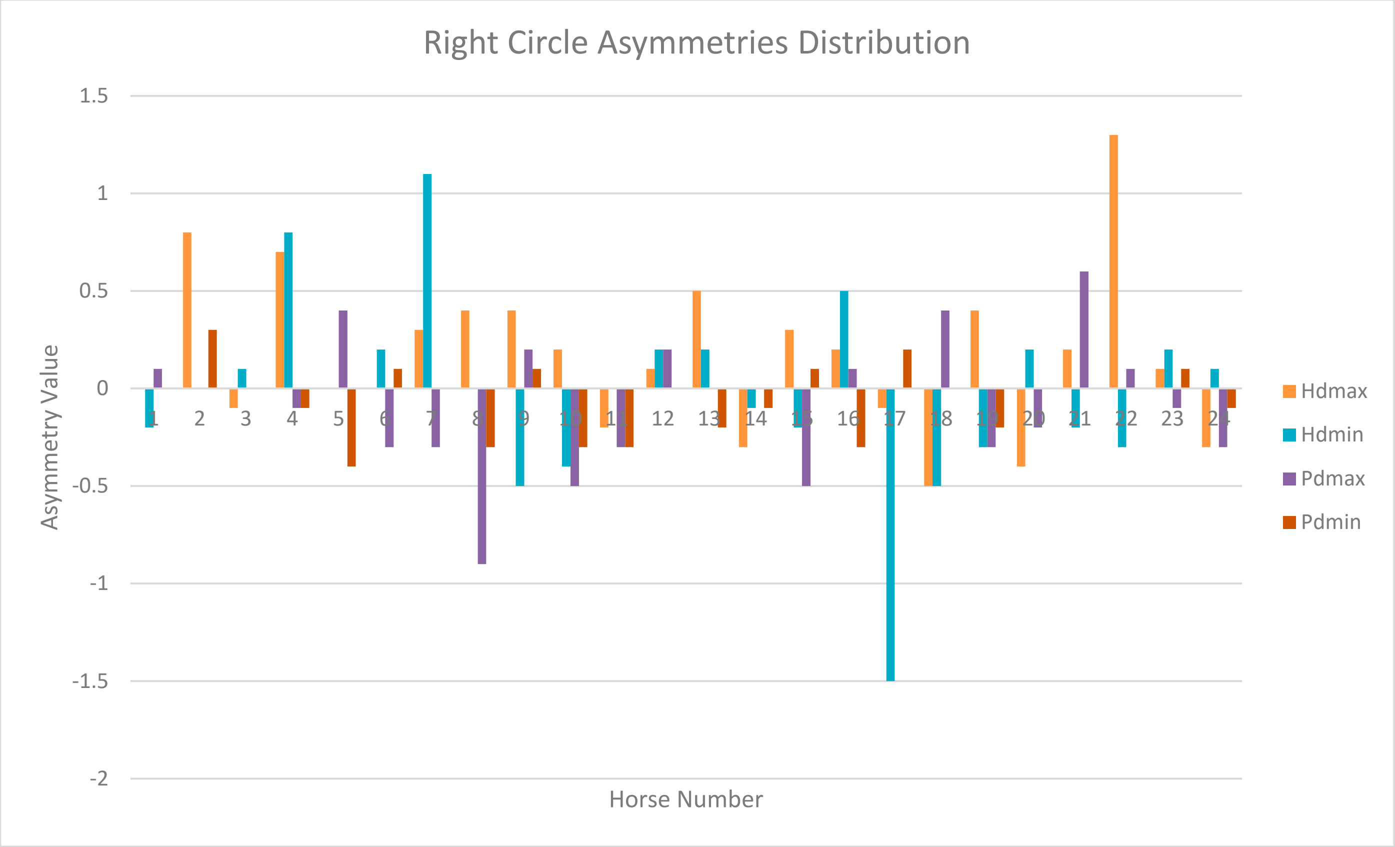

Figure S2. Vertical displacement asymmetry parameters during right-circle lungeing for each horse (n = 24). Bar plots show the values of HDmin, HDmax, PDmin, and PDmax for each individual horse, as measured during right circle lungeing. All horses included in the study are shown, and all asymmetry values (including those below the clinical threshold) are presented. No data are missing; values close to zero may appear small or indistinct on the plot.

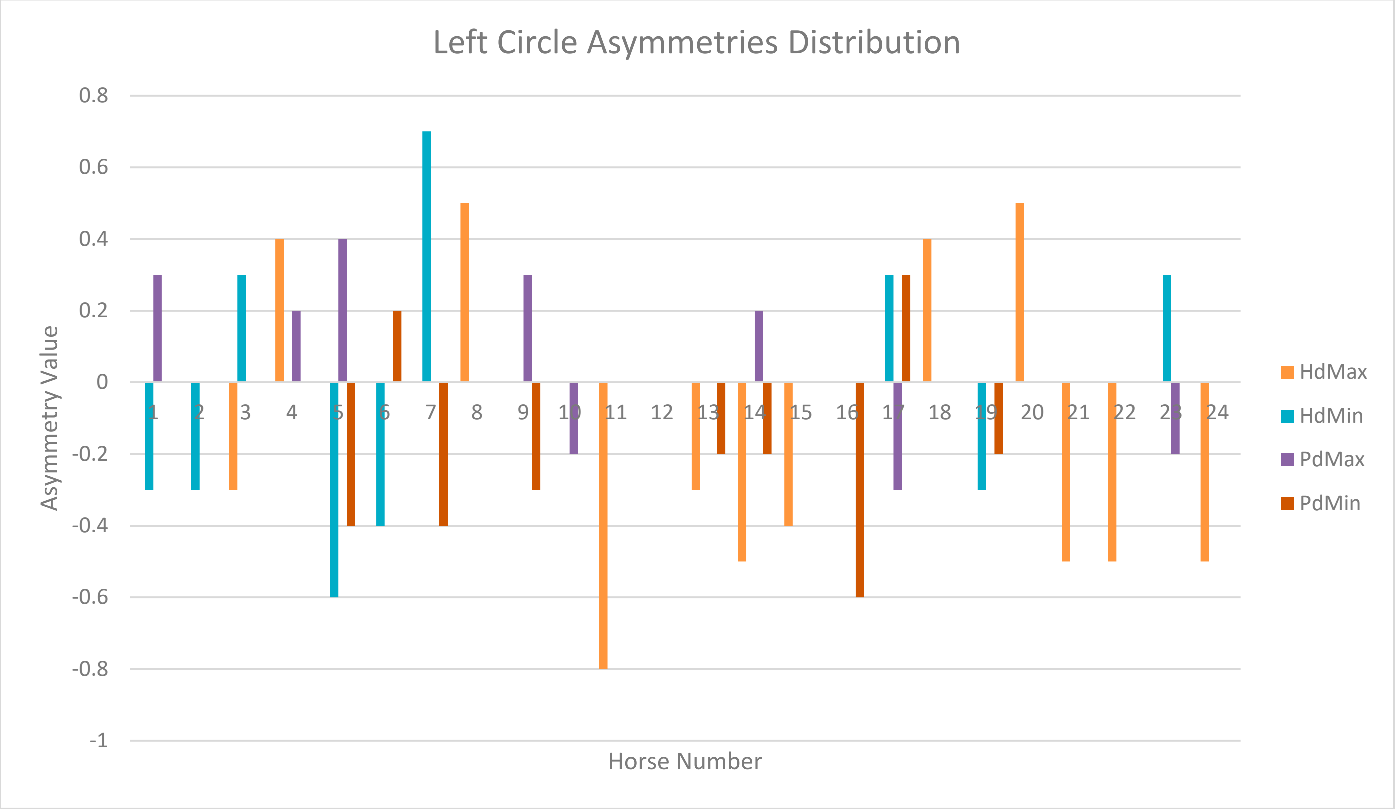

Figure S3. Vertical displacement asymmetry parameters during left-circle lungeing for each horse (n = 24). Bar plots show the values of HDmin, HDmax, PDmin, and PDmax for each individual horse, as measured during left circle lungeing. All horses included in the study are shown, and all asymmetry values (including those below the clinical threshold) are presented. No data are missing; values close to zero may appear small or indistinct on the plot.

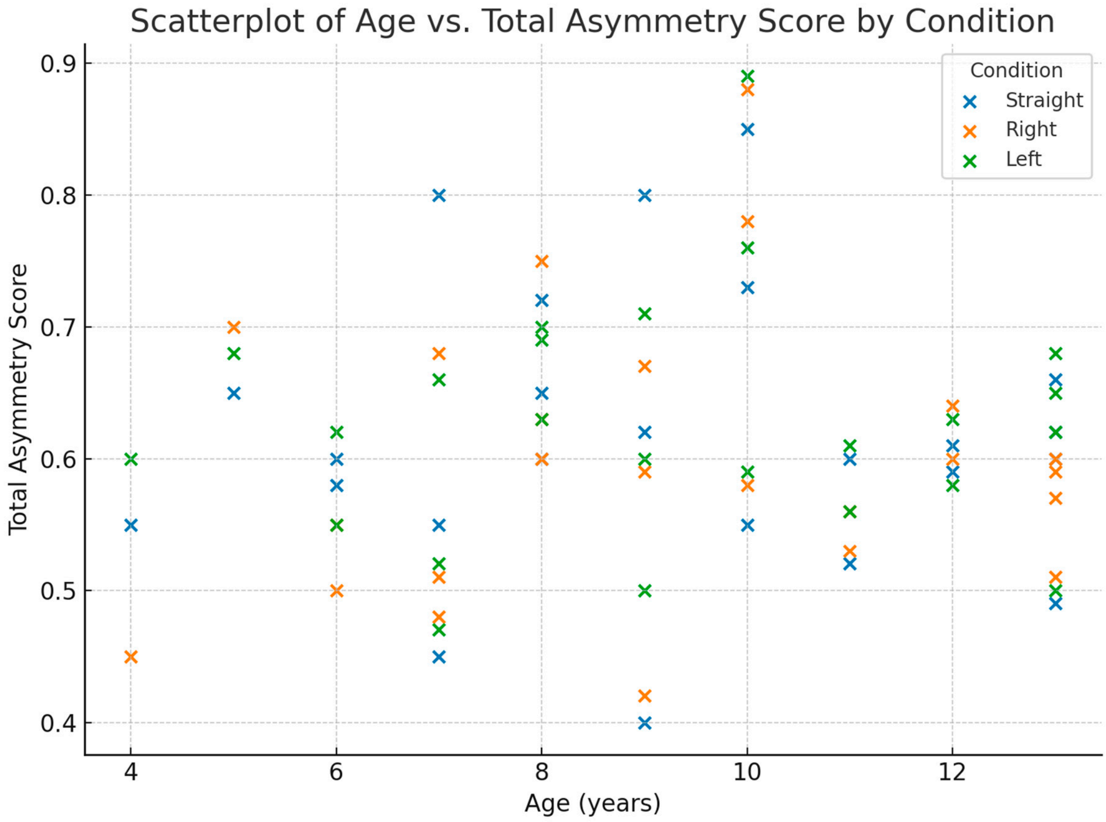

Figure S4: Scatterplot of horse age versus total asymmetry score across movement conditions (n = 24).

Each point represents the total asymmetry score of a horse in a given movement condition (straight line, right-circle lungeing, or left-circle lungeing).

Points are color-coded by condition: blue for straight-line, orange for right-circle, and green for left-circle evaluations.

No evident correlation, trend, or condition-specific clustering was observed, supporting the absence of a statistical association between age and total asymmetry scores in this cohort.
